# Using First Principles for Deep Learning and Model-Based Control of Soft Robots

**DOI:** 10.3389/frobt.2021.654398

**Published:** 2021-05-04

**Authors:** Curtis C. Johnson, Tyler Quackenbush, Taylor Sorensen, David Wingate, Marc D. Killpack

**Affiliations:** ^1^Robotics and Dynamics Lab, Department of Mechanical Engineering, Brigham Young University, Provo, UT, United States; ^2^Perception, Control, and Cognition Lab, Department of Computer Science, Brigham Young University, Provo, UT, United States

**Keywords:** deep learning, model predictive control, soft robots, error modeling, data-driven modeling, dynamics

## Abstract

Model-based optimal control of soft robots may enable compliant, underdamped platforms to operate in a repeatable fashion and effectively accomplish tasks that are otherwise impossible for soft robots. Unfortunately, developing accurate analytical dynamic models for soft robots is time-consuming, difficult, and error-prone. Deep learning presents an alternative modeling approach that only requires a time history of system inputs and system states, which can be easily measured or estimated. However, fully relying on empirical or learned models involves collecting large amounts of representative data from a soft robot in order to model the complex state space–a task which may not be feasible in many situations. Furthermore, the exclusive use of empirical models for model-based control can be dangerous if the model does not generalize well. To address these challenges, we propose a hybrid modeling approach that combines machine learning methods with an existing first-principles model in order to improve overall performance for a sampling-based non-linear model predictive controller. We validate this approach on a soft robot platform and demonstrate that performance improves by 52% on average when employing the combined model.

## 1. Introduction

Soft robots have many desirable characteristics which make them attractive candidates for a wide variety of tasks where traditional rigid robots are ill-suited. For example, rigid robots are often restricted to operating in well-defined enclosures to avoid dangerous collisions with the environment or human operators. In contrast, soft robots are able to operate safely in unstructured environments, where incidental contact is likely or even desired, due to their inherent flexibility and adaptability.

In this work, the main contribution we present is a methodology for learning model discrepancies for use in a real-time non-linear model predictive control (NMPC) scheme. We validate this approach in simulation and on a soft robot platform. This platform is an ideal test bed for our approach because the actual dynamics (both in terms of joint configuration and air pressure in the joint chambers over time) are intrinsically more uncertain than previously presented rigid robot systems and control methods discussed in section 1.1. While we apply our approach to soft robotics to demonstrate its potential to learn both uncertain and unknown dynamics, the proposed method could generalize to any platform using a model predictive controller.

The structure of this paper is as follows. Section 2 presents our hardware platform, the analytical model used to generate training data, our deep neural network (DNN) training methods, and evaluation of each model's accuracy. Section 3 explains the non-linear evolutionary model predictive control (NEMPC) algorithm we employ and shows the results of our experiments and explores their implications. Section 4 discusses the importance of this work as well as current limitations and future directions for additional research.

### 1.1. Related Work

The many desirable characteristics of soft robots present challenging problems when it comes to modeling and controlling them. Accurate physics-based (first-principles) models that are tractable for real-time model-based control are difficult to obtain because of uncertain material properties, hysteresis, non-linear dynamics, and complicated pneumatic flow dynamics. Soft robot physics-based modeling efforts range from finite element (FEM) approaches as in Pozzi et al. (2018) and Katzschmann et al. (2019) to Cosserat Rod models as in Till et al. (2019) or piecewise constant curvature (PCC) models as in Allen et al. (2020) and Della Santina et al. (2020). Many of these methods have shown promise. However, the effort and expertise required to accurately model all of the aforementioned effects is formidable. Even if a perfectly accurate analytical model could be derived, it may be useless for real-time model-based control due to the high computational time required for evaluation, as will be shown in the experiments of section 3.5. Additionally, even if the model is made tractable using appropriate simplifications, it would likely still require significant effort in system identification to obtain acceptable closed loop control performance.

Rus and Tolley (2015) and Thuruthel et al. (2018) both summarize the wide spectrum of strategies that have been proposed to overcome the aforementioned modeling challenges. Among these, data-driven modeling specifically addresses many difficulties of physics-based modeling for control. Generally, data-driven control algorithms are based on various forms of machine learning, such as neural networks as in Thuruthel et al. (2017) and Mohajerin et al. (2018), Gaussian processes (GP) in Ostafew et al. (2016), Kabzan et al. (2019), Soloperto et al. (2018), and Hewing et al. (2020), reinforcement learning (RL) as in Thuruthel et al. (2019), or sparse optimization (also known as SINDY) as in Kaiser et al. (2018). Notably, deep learning has proven to be a valuable tool for robot modeling and control and is explored thoroughly in Pierson and Gashler (2017) and Sünderhauf et al. (2018). Deep learning has more recently demonstrated the ability to approximate soft robot dynamic models accurately in Gillespie et al. (2018) and Hyatt and Killpack (2020). A major benefit of such approaches is that they are largely data-driven and as such, do not require an analytical model or specialized expertise. However, using these learned models in a real-time, model-based control formulation for soft robots (such as in Gillespie et al., 2018; Hyatt and Killpack, 2020) has been explored to a much lesser extent. Specifically, by using specialized hardware for accelerated computing, such as Graphics Processing Units (GPUs), data-driven models can be forward sampled in large batches and at high rates using a parallelized architecture. This enables their direct use to solve an optimal control problem using a non-linear model predictive control strategy (see Hyatt and Killpack, 2017; Hyatt et al., 2020b). This is the approach on which we build for this paper.

On the other hand, an undesirable characteristic of data-driven modeling techniques is the need for large amounts of representative data, which is difficult to collect on hardware platforms where exploring the whole state space of the robot is infeasible or dangerous. Our approach in this paper is to use a simplified, first-principles model to train a deep neural network (DNN) to represent general trends in state variables for the dynamics, and then add another deep neural network to compensate for additional error in the predicted states. To accomplish this, while also benefiting from the parallel computation available on a GPU, we first train a DNN to learn the first-principles model. Then we train a second DNN to learn the simulation-to-reality error gap. Because the first-principles DNN learns the general form of the dynamics from simulation, much less hardware training data is required. The hardware data only serves to make adjustments to capture unmodeled dynamics and does not necessarily need to be as representative or as plentiful as would be required if hardware data was exclusively used to train the neural network.

Our work toward compensating for modeling error with data-driven learning is similar to Sun et al. (2019) where authors use deep learning to predict physics-based modeling error of water resources, Kaheman et al. (2019) where they present an algorithm to learn a discrepancy model on an double inverted pendulum, and Della Santina et al. (2020) where the authors augment a model-based disturbance observer with a learned correction factor on a soft robot. Most similar to our work is that of Koryakovskiy et al. (2018) where they augment a non-linear model predictive controller with various forms of learned actions to compensate for model-plant mismatch on a rigid humanoid robot. Other works that include using neural networks as the backbone for predictive control are Piche et al. (2000) and Lu and Tsai (2008).

## 2. First Principles and Deep Learning

We start by providing an overview of our approach and how it fits with the methods and hardware presented in subsequent sections. Our overall approach to compensate for unknown modeling errors starts with training a deep neural network to act as a surrogate for the analytical model derived in section 2.1. This surrogate DNN is needed to exploit the parallelized architecture of modern GPUs, which in turn, affords higher control rates for our non-linear MPC algorithm described in section 3.1. Details related to the training of the surrogate DNN are presented in section 2.2.

Next, we train a second deep neural network to compensate for modeling discrepancies described in section 2.3. The methods for training this error DNN are presented in section 2.4.

Once the surrogate and the error DNN are trained we evaluate both in parallel, resulting in a combined forward prediction model (that we refer to as a combined DNN) which reflects the dynamics of the hardware platform more accurately. By improving the forward prediction capabilities of our model, we enable the controller to find more optimal input trajectories and thereby improve control performance. The methods involved in validating the control performance using the combined DNN are presented in section 3.4.

Data, code, models, and dynamic parameters are available at https://github.com/BYU-PCCL/DL-MPC.

### 2.1. Robot Platform Description and Modeling

**Figure 1:**
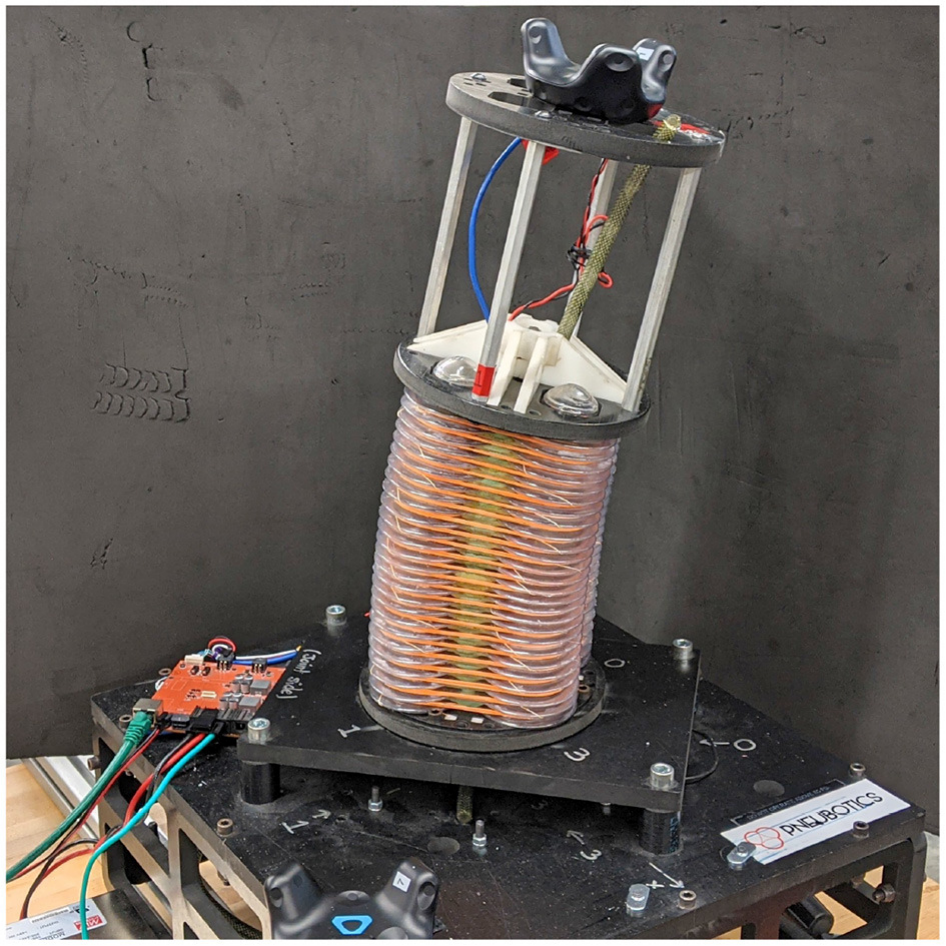
Photograph of soft robotic continuum joint used for this work. θ and ϕ are the rotations about the joint's x and y axes, respectively.

The platform used for this work is a continuum joint comprised of four pressurized bellows which encircle an inextensible steel cable, as shown in Figure 1. Controlling the pressure in each of the bellows results in a net torque which causes the joint to bend. We use the same singularity-free kinematic relationships derived by Allen et al. (2020) where the curvature of the continuum joint is parameterized as two separate rotations (*u* and *v*) about orthogonal axes (*x* and *y*), which lie at the base of the joint. For notational clarity in this paper, we define θ = *u* and ϕ = *v*.

The dynamic model of the continuum joint is of the form

(1) $$\begin{array}{l} M\left(q\right)\ddot{q}+C\left(q,\overset{∙}{q}\right)\overset{∙}{q}+g\left(q\right)=\tau \end{array}$$

where *M*(*q*) ∈ ℝ^2×2^ is the symmetric mass matrix, $C\left(q,\overset{∙}{q}\right)\in {\mathbb{R}}^{2\times 2}$ is the Coriolis matrix, *g*(*q*) ∈ ℝ^2^ is a vector of torques caused by gravity, *q*(*t*) = [θ, ϕ]^⊤^ is a vector of generalized coordinates, and τ ∈ ℝ^2^ is a vector of generalized forces.

An analytical equation of motion of the form shown in Equation (1) can be derived using principles of Lagrangian mechanics by modeling the joint as an infinite set of infinitesimally thin disks and integrating along the length of a piecewise constant curvature (PCC) arc. This method was developed in Hyatt et al. (2020a), which includes a detailed derivation of this model.

There are also significant non-linear pressure dynamics inside of the bellow actuators, where the rate of change in pressures is on the same order of time response as the actual motion of the robot. We model the pressure dynamics as a first-order system such that

(2) $$\begin{array}{l} ṗ\left(t\right)=\alpha \left(p_{ref}\left(t\right)-p\left(t\right)\right) \end{array}$$

where *p*(*t*) ∈ ℝ^4^ is a vector of pressures, $p_{ref}\left(t\right)\in {\mathbb{R}}^{4}$ is a vector of reference (i.e., commanded) pressures, and α ∈ ℝ^4×4^ is a diagonal matrix of coefficients representing the fill/vent rate of the pneumatic valves. Numerical values for the parameters used in this model are included in the repository accompanying this paper.

Because each of the pressure bellows is made of deformable plastic, there are several effects from material properties, such as stiffness and damping that are not accounted for in Equation (1). We include these effects as a linear spring term (*K_spring_q*, where *K_spring_* is a diagonal matrix), which pulls the joint toward a completely vertical configuration, and a viscous damping term ($K_{d}\overset{∙}{\text{q}}$, where *K_d_* is also a diagonal matrix). The pressure-to-torque mapping term (*K_prs_p*) maps pressure differentials in each antagonistic pair of bellows to a torque about each axis where bending in ϕ and θ occur. These additions, coupled with Equation (2), result in our final analytical dynamic model:

(3) $$\begin{array}{l} M\left(q\right)\ddot{q}+C\left(q,\overset{∙}{q}\right)\overset{∙}{q}+g\left(q\right)=K_{prs}p-K_{d}\overset{∙}{q}-K_{spring}q \end{array}$$

For conciseness, we rearrange Equations (2) and (3) into a non-linear state variable form

(4) $$\begin{array}{l} ẋ\left(t\right)=\left(\begin{array}{ccc} -\alpha & 0 & 0\\ M^{-1}K_{prs} & M^{-1}\left(-K_{\text{d}}-C\right) & -M^{-1}K_{spring}\\ 0 & I & 0 \end{array}\right)\\ \left(\begin{array}{c} p\\ \overset{∙}{q}\\ q \end{array}\right)+\left(\begin{array}{c} \alpha \\ 0\\ 0 \end{array}\right)p_{ref}\left(t\right)-M^{-1}g \end{array}$$

where $x\left(t\right)\equiv {\left[p,\overset{∙}{q},q\right]}^{⊤}$ and *u*(*t*) ≡ *p_ref_*(*t*). We use *x*(*t*) and *u*(*t*) for the remainder of this work.

### 2.2. Surrogate DNN Training

We first train a neural network to learn a state transition function from simulated data using the analytical model described in Equation (4). Previous work in the field of reinforcement learning (OpenAI et al., 2019; Rao et al., 2020) demonstrated that using simulated data, while not reflecting the world perfectly, allows the model to learn more quickly and perform better than when the model is trained on real-world data alone. Leveraging analytical models and simulation environments also allows us to be able to collect more data than would be physically possible since simulations can run for long periods of time without supervision and without the risk of damaging hardware. In simulation, data is cheap and easy to collect. With the only cost for collecting data being computing power and time, we theoretically have access to an infinite dataset without risking any damage to the real robot hardware.

Training data is generated by numerically integrating Equation (4) with a fourth order Runge-Kutta integration scheme using a constant time step of 0.001 s in order to get accurate simulation data. The pressure commands (*u*(*t*)) are square waves randomly distributed between the minimum and maximum safe operating pressures (8–400 kPa) in order to record both transient and steady-state responses for DNN training. We use square waves because of their ability to excite (and therefore learn) more dynamic modes in the system compared to other common test signals (e.g., sine waves or ramps). The simulated training data consists of 12 simulation runs, each over a period of 250 s. Sampled at a rate of 0.001 s, this came out to three million data points.

We frame the training process as a supervised learning problem, with the current state (*x*_*t*_) and commanded pressures (*u*_*t*_) being inputs, and the difference between the current state and the next state (Δ*x*_*t*_ = *x*_*t*+1_ − *x*_*t*_) as the output. Since the changes in state are small over small time steps, by only requiring the model to learn the difference in states, we free the model from mostly having to learn the identity operation of copying over the previous state with only small adjustments.

**Figure 2:**
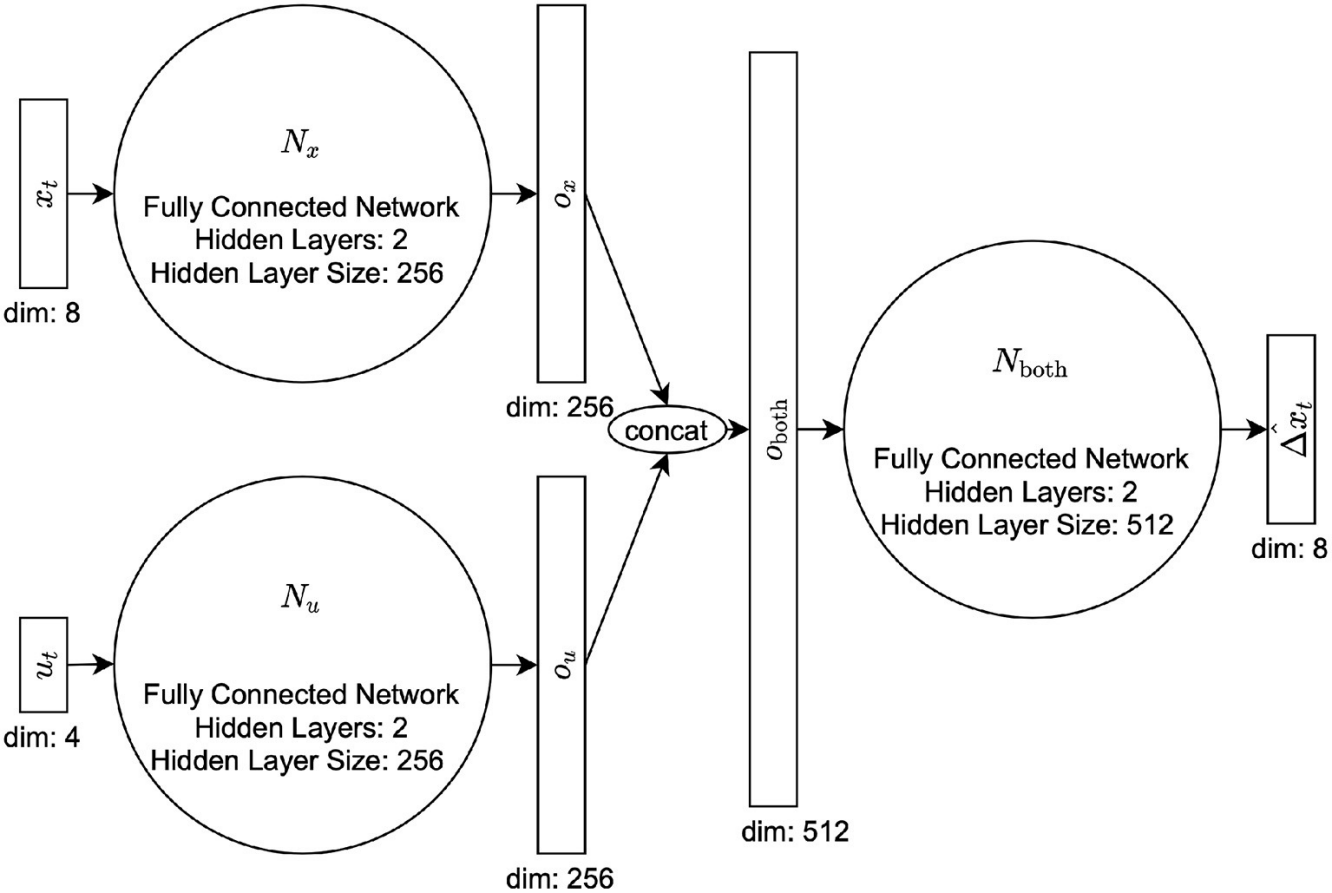
DNN Architecture, used for both the surrogate model and the error model.

In training, we use a simple fully-connected network. In situations where accuracy is desired more over speed, one might instead opt for a long short-term memory (LSTM, Hochreiter and Schmidhuber, 1997) or transformer neural architecture (Vaswani et al., 2017). However, we chose to use this small, simple network to allow for the very quick evaluation time that is needed for NEMPC. Each network is composed of three intermediate fully-connected networks which we call *N*_*x*_, *N*_*u*_, and *N_out_*. All hyperparameters, including the number of hidden layers and hidden layer sizes, were chosen using a hyperparameter search while maintaining the speed necessary for real-time control (see Table 1 for full list of parameters). *N*_*x*_ and *N*_*u*_ are fully-connected networks with two hidden layers, and 256 hidden nodes. *N_out_* has two hidden layers and 512 hidden nodes. For context, we let the state and network outputs be $x_{t},\Delta x_{t}\in {\mathbb{R}}^{8}$, and let the commanded pressure be $u_{t}\in {\mathbb{R}}^{4}$. We run *x*_*t*_ through *N*_*x*_ and *u*_*t*_ through *N*_*u*_ to produce intermediate outputs $o_{x}\in {\mathbb{R}}^{256}$ and $o_{u}\in {\mathbb{R}}^{256}$, respectively. The two intermediate outputs are concatenated [$o_{both}\in {\mathbb{R}}^{512}=concatenate\left(o_{x},o_{u}\right)$] and run through *N_out_* to produce the state transition from the current time step to 0.02 s (the prediction rate of the controller) in the future, $\Delta {\hat{x}}_{t}=x_{t+1}-x_{t}$. Because our data was recorded at 1,000 Hz, we had to sample from our training data at the correct frequency of 50 Hz (taking every twentieth data point) to help the network learn at the desired control rate of 50 Hz. We use 70% of the data for training and reserve 30% for validation. For a diagram of the architecture, please refer to Figure 2. We calculate the loss to be the L1-norm plus the cosine distance.

(5) $$\begin{array}{l} l=||x_{t}-{\hat{x}}_{t}{||}_{1}+c\left(x_{t},{\hat{x}}_{t}\right) \end{array}$$

where cosine distance (*c*) is

(6) $$\begin{array}{l} c\left(x_{t},{\hat{x}}_{t}\right)=1-\frac{x_{t}\cdot {\hat{x}}_{t}}{||x_{t}|{|}_{2}||{\hat{x}}_{t}|{|}_{2}} \end{array}$$

We chose this loss in order to account for both the total absolute error and the direction of change. This direction of change matters because if the predicted rate of change in our state has the wrong sign, this can cause significant stability problems for model-based control. Note that we normalize *x*_*t*_ and *u*_*t*_ to have a mean of zero and standard deviation of one before running them through *N*_*x*_ and *N*_*u*_ to allow for faster training. The difference between states Δ*x*_*t*_ = *x*_*t*+1_ − *x*_*t*_ is scaled by the standard deviation before calculating the loss function to allow the loss function to weight all state variables equally, regardless of the unit, but is not shifted by the mean to preserve direction. At evaluation time, we re-scale the derivative to the correct units with our cached standard deviations.

**Algorithm 1:** Surrogate DNN Training Procedure

1: **for** *epoch* = 1 to *NumEpochs* **do**
2: **for** each simulated training sequence of length *n* **do** ⊳ Around 2 million training sequences
3: Let *x*_0_, *x*_1_, ..., *x*_*n*_ be sequence of states ⊳ Each *x*_*i*_ is a vector of size 8
4: Let *u*_0_, *u*_1_, ..., *u*_*n*−1_ be sequence of commanded pressures ⊳ Each *u*_*i*_ is a vector of size 4
5: ${\hat{x}}_{1}=N_{sim}\left(x_{0},u_{0}\right)+x_{0}$ ⊳ Only allow *DNN* to see first true state
6: $l=||x_{t}-{\hat{x}}_{t}|{|}_{1}+c\left(x_{t},{\hat{x}}_{t}\right)$ ⊳ Calculate loss
7: **for** *i* = 1 to *n* − 1 **do**
8: ${\hat{x}}_{i+1}=N_{sim}\left({\hat{x}}_{i},u_{i}\right)+{\hat{x}}_{i}$ ⊳ Use previous estimated state to propagate loss/error
9: $l=l+||x_{t}-{\hat{x}}_{t}|{|}_{1}+c\left(x_{t},{\hat{x}}_{t}\right)$ ⊳ Add loss at each time step
10: **end for**
11: Backpropagate *l* ⊳ Backpropagate total loss
12: Update *N_sim_* weights with Adam optimizer
13: **end for**
14: **end for**

**Table 1:** Hyperparameters used for training each type of DNN.

	**Data**	**Hidden layers**	**Hidden sizes**	**Learning rate**	**Activation**	**Dropout**
*N_sim_*	Simulated	2, 2	256, 512	1.89e-4	Leaky ReLU	0.396
*N_err_*	Hardware	2, 2	256, 512	7.99e-4	Leaky ReLU	0.396

For non-linear model predictive control (NEMPC), or any other predictive control algorithm, we need to be able to accurately predict more than just one time step ahead. To make our DNN more robust and accurate across longer time intervals, we do not train the network to only predict one time step into the future. Instead, we only allow the network to see the first state in a sequence (like an initial condition for numerical integration). Then using the pressure inputs, we train the network to estimate *n* steps forward by recursively running the estimated states through the network. Note that we backpropagate the total loss over the entire trajectory at each training step. By training this way, we can be more confident that any unmodeled error will not propagate forward in time. When choosing the number of steps (*n*) for forward propagation of the dynamic model, one should consider the desired horizon where we need the most accurate predictions. We chose *n* = 100 so that our model would be able to predict 2 s (100 · 0.02 s) into the future–a horizon longer than most that would be used with NEMPC–as detailed in section 3 (For reference, the time horizon we use in this work for real-time control is 0.1 s).

We train the surrogate DNN on data gathered from the analytical model described in section 2.1. For a detailed description of the training procedure, please refer to Algorithm 1. Note that we refer to the surrogate DNN as *N_sim_*.

### 2.3. Dynamic Model Inaccuracies

In this section we discuss in more detail the modeling errors and partially correct assumptions that exist in the dynamic model presented in section 2.1 in an attempt to understand and gain intuition as to how the trained error model will compensate during real-time control.

Regarding Equation (2), we acknowledge that the real pressure dynamics on hardware are not simply first order. For example, we do not model the dynamics of the valves used to control pressures (which can cause choked or unchoked fluid flow) and the differences in the pressure dynamics depending on whether the chambers are filling or venting from different pressure reservoirs. In Equation (3), we assume a linear pressure to torque mapping (*K_prs_p*), a linear damping term ($K_{d}\overset{∙}{q}$), and a linear spring term (*K_spring_q*). These terms do not capture non-linear behaviors, such as increased stiffness and damping that exist near joint limits, nor do they reflect any wear in the materials due to usage over time. We also suspect some hysteresis in the movement of the joint, as well as an offset in the resting equilibrium position for ϕ and θ of the robot due to plastic deformation in each of the robot's pressure chambers. None of these previous effects are explicitly included in the dynamic model of the robot.

While previous work (Hyatt et al., 2020a) demonstrated that this formulation of the dynamic model was accurate enough for model-based control, improvements are needed in order to control soft robots in uncertain environments or during highly dynamic movements. Certainly, further system identification would improve this model; however, because of the complexities and uncertainties inherent in soft robots and the processes to manufacture them, system identification techniques scale poorly with high degree-of-freedom systems and do not necessarily generalize well between platforms. The error model developed in this paper offers a scalable technique to compensate for modeling error while still maintaining generality between platforms.

### 2.4. Error DNN Training

**Figure 3:**
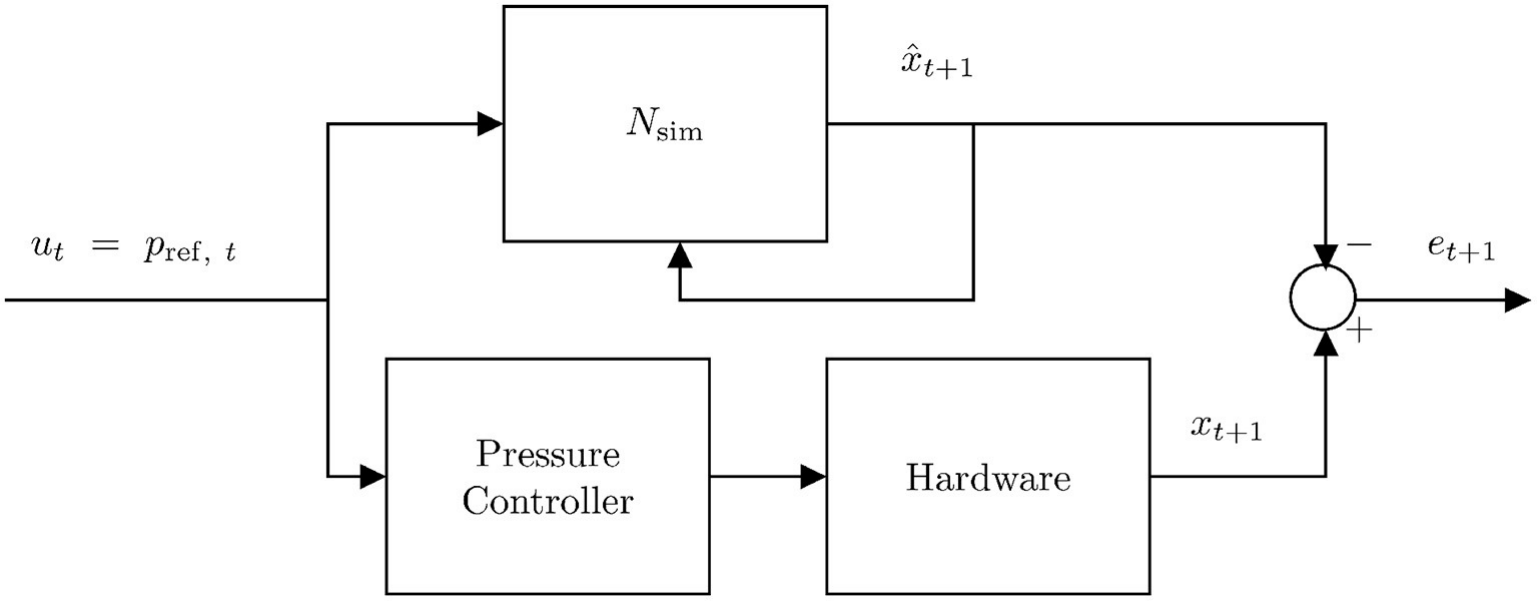
This diagram shows the method used to generate error training data. A random step input pressure trajectory *u* is sent to the hardware and the states are recorded. The same input trajectory is simulated using the surrogate DNN which takes *u* and *x* as inputs. The resulting state trajectory is subtracted from the hardware state trajectory to get the state tracking error over time. This allows the error DNN to predict error given the current state *x* and the current input *u*.

To train an error model that is capable of compensating for the model inaccuracies described in section 2.3, we first collect hardware data by sending and recording bounded random pressure inputs *u*(*t*) to the robot. The robot's internal pressure controller ensures that the pressure in each of the bellows reaches the commanded pressure. Note that the minimum and maximum safe operating pressures (8–400 kPa) are also respected here through external pressure regulation. We record the joint positions and pressures directly and estimate joint velocities numerically. This process is repeated for each time step until a suitable quantity of training data is gathered (see Figure 3). By nature, this hardware data is noisy and inconsistent, with sampling rates varying slightly during the data collection process. In order to train on data with uniform spacing, we interpolate between real data points to estimate the state vector and inputs at regular 0.001 s intervals. We trained with more simulated data than hardware data, with only seven hardware runs which are each 90 s long, coming out to 630,000 data points. We use 540,000 data points for training and 90,000 to validate the model.

With the data gathered from the hardware, we were able to train the error DNN. First, we sample the data at the desired time interval for which the surrogate model was trained (0.02 s). We then freeze the weights of the surrogate DNN, and divide our dataset into sequences of length *n* = 100. In a similar training procedure as before, we only allow the network to see the first state *x*_0_, and task it with predicting the next *n* states given the commanded pressures *u*_0_, *u*_1_, ..., *u*_*n*−1_. We run the states through the surrogate DNN, and add to its output the output of the error DNN. Thus, the error model does not have to learn the first-principle physics that the surrogate DNN has already learned. Instead, it only has to learn the discrepancies between the simulation and reality, as discussed in section 2.3. We pass the first state and sequence of commanded pressures *n* times recursively through both the surrogate and error networks, calculate loss between the true and predicted error, and update the error network's weights (see Algorithm 2). We use 80% of the data for training and reserve 20% for validation. Note that we refer to this error DNN as *N_err_*. When convergence is reached, both DNN models are ready to be utilized within NEMPC for forward prediction of the robot's behavior.

### 2.5. Modeling Results

**Figure 4:**
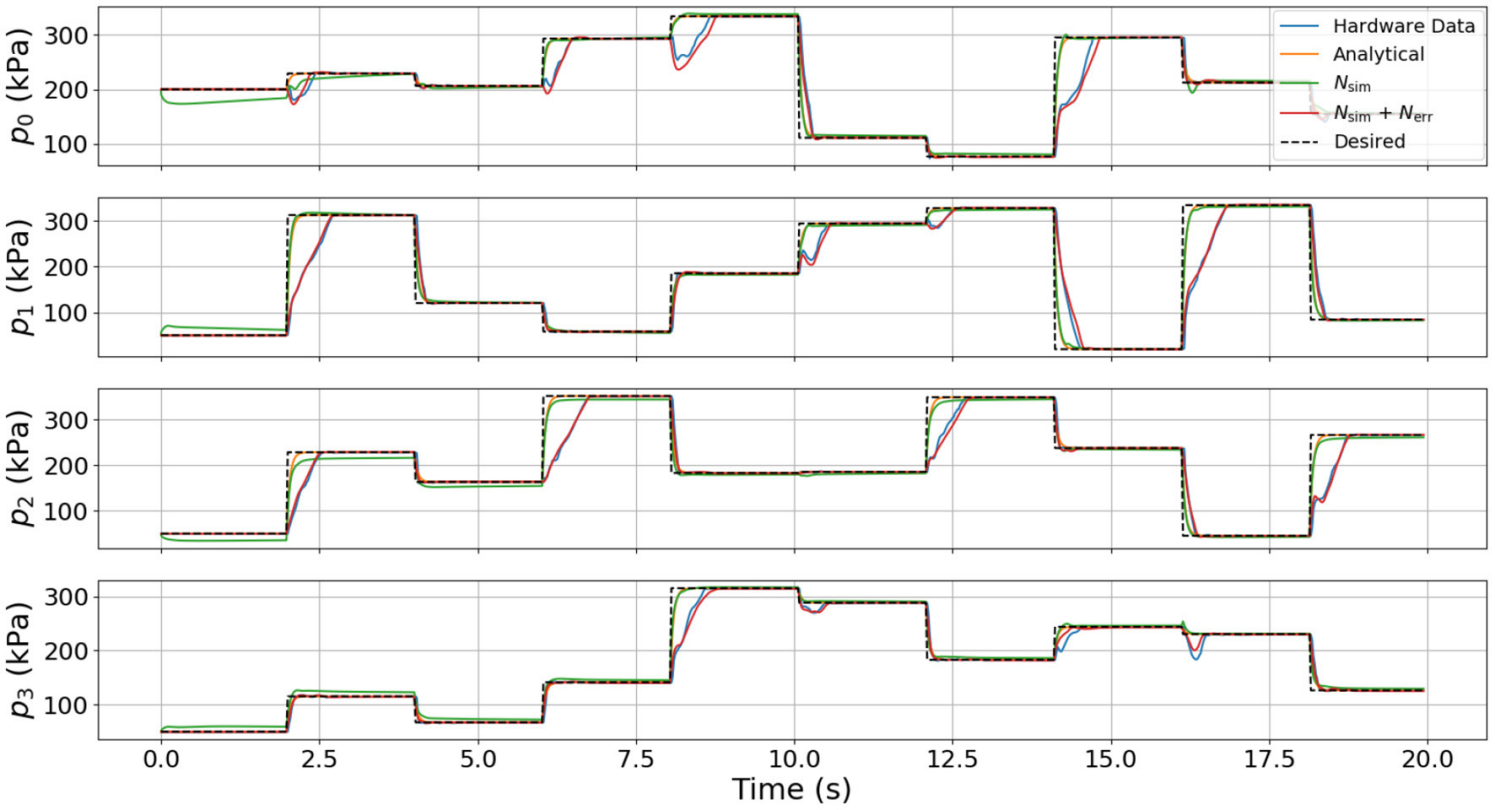
Comparison of pressure dynamics between the four different systems used. The dashed line indicates the commanded pressure, while each of the solid lines is the pressure response resulting from the commanded pressure input. Note that the states from the analytical model and the surrogate DNN match well and that when using the combined DNN, the simulation closely resembles the hardware data.

**Figure 5:**
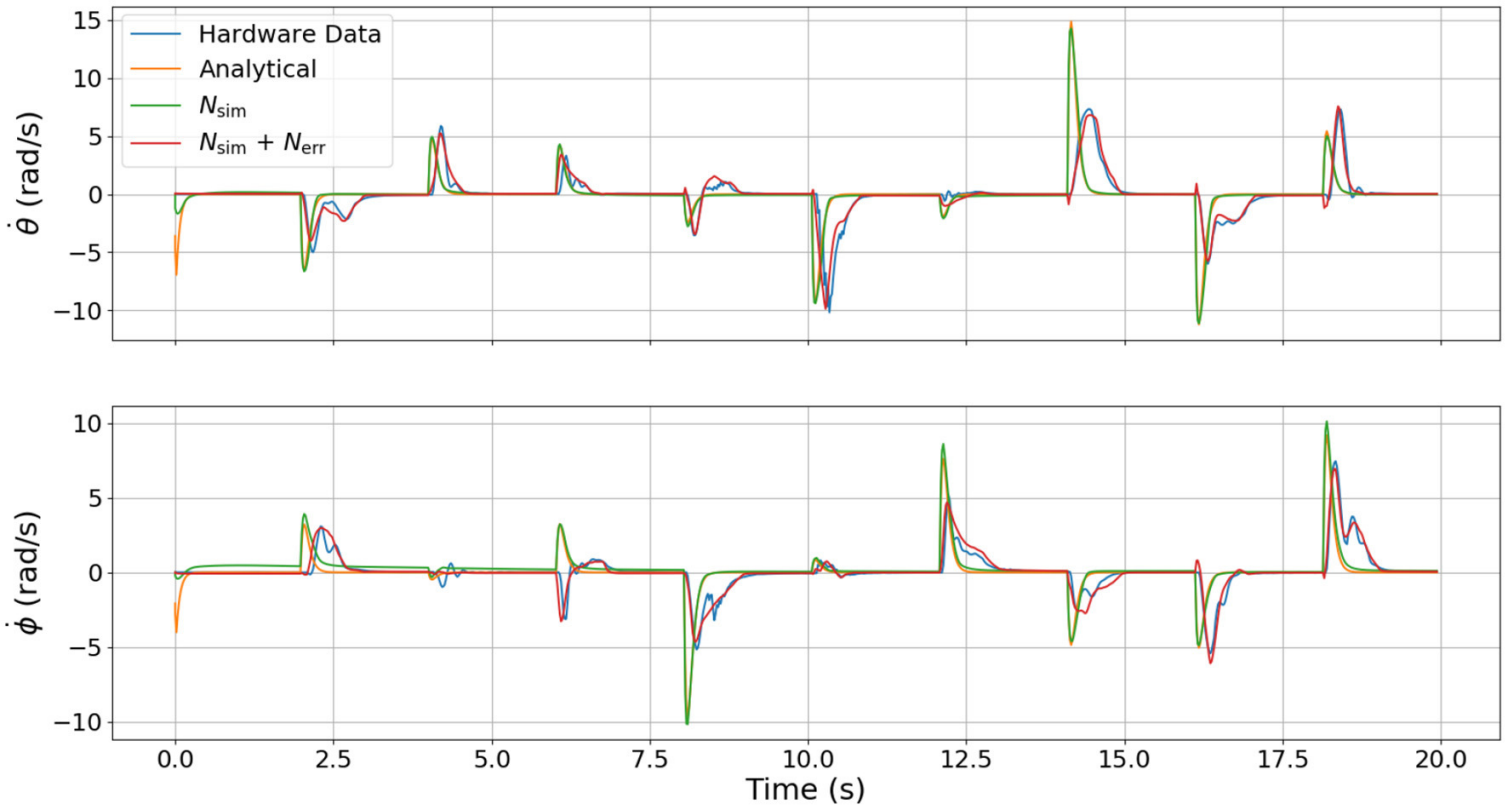
Comparison of velocity dynamics between the four different models used. These velocities are the response resulting from the commanded pressure inputs shown in Figure 4. Note that the surrogate DNN tracks the analytical model well while the combined DNN tracks the hardware data well.

**Figure 6:**
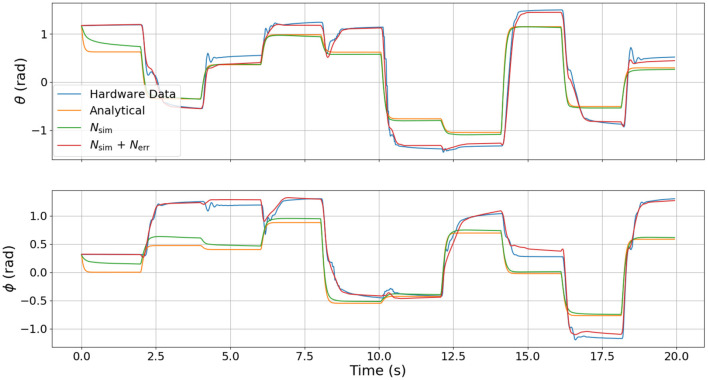
Comparison of joint angle dynamics between the four different models used. These angles are the response resulting from the commanded pressure inputs shown in Figure 4.

To test the relative fidelity of each model and compare their responses, we simulate the analytical model, the surrogate DNN, and the combined DNN (i.e., the surrogate DNN plus the error DNN or *N_sim_* + *N_err_*) with a random step trajectory of commanded pressures (*u*(*t*)). This same pressure trajectory is then also commanded on hardware to enable a complete comparison between all models and the actual hardware platform. Figures 4–6 compare the dynamic response of the four different systems (e.g., analytical model, *N_sim_*, *N_sim_* + *N_err_*, actual hardware) in pressure, angular velocity, and joint angles, respectively. It is important to note that while there is significant steady-state error as well as some unmodeled transients in all three figures, the analytical model captures the general trends of the hardware data. Because these trends are naturally embedded in the training data, the error DNN is only required to learn small adjustments which requires much less data than learning the dynamics from scratch. Additionally, in all three figures, the surrogate DNN tracks the analytical model with relatively small error. This indicates that the surrogate DNN training was successful. Because the surrogate DNN is trained with simulated data, it could easily be improved further by running more simulations. Likewise, the combined DNN tracks the hardware data well.

**Algorithm 2:** Error DNN Training Procedure

1: Freeze *N_sim_* weights
2: **for** *epoch* = 1 to *NumEpochs* **do**
3: **for** each hardware training sequence of length *n* **do** ⊳ About 540 thousand training sequences
4: Let *x*_0_, *x*_1_, ..., *x*_*n*_ be sequence of states
5: Let *u*_0_, *u*_1_, ..., *u*_*n*−1_ be sequence of commanded pressures
6: ${\hat{x}}_{1}=N_{err}\left(x_{0},u_{0}\right)+N_{sim}\left(x_{0},u_{0}\right)+x_{0}$ ⊳ Add *N_err_* output to simulation model, learn the gap
7: $l=||x_{t}-{\hat{x}}_{t}|{|}_{1}+c\left(x_{t},{\hat{x}}_{t}\right)$
8: **for** *i* = 1 to *n* − 1 **do**
9: ${\hat{x}}_{i+1}=N_{\text{err}}\left({\hat{x}}_{i},u_{i}\right)+N_{sim}\left({\hat{x}}_{i},u_{i}\right)+{\hat{x}}_{i}$
10: $l=l+||x_{t}-{\hat{x}}_{t}|{|}_{1}+c\left(x_{t},{\hat{x}}_{t}\right)$
11: **end for**
12: Backpropagate *l*
13: Update *N_err_* weights with Adam optimizer
14: **end for**
15: **end for**
16: Define *DNN* such that *DNN*(*x*_*t*_, *u*_*t*_) = *N_sim_*(*x*_*t*_, *u*_*t*_)+*N_err_*(*x*_*t*_, *u*_*t*_)

It is clear from Figure 4 that the error DNN learned that the actual pressure dynamics on hardware are not first order as is predicted by the analytical and surrogate DNN. We believe these differences arise from valve/flow dynamics when venting or filling a pressure chamber aggressively. The most salient feature in Figure 5 is that the velocities on hardware tend to lag behind those of the analytical model due to the filtering of measured position data, which introduces a small phase lag. Interestingly, the combined DNN still tracks the hardware data well, revealing a promising ability to compensate for errors introduced not only by modeling error, but also by state estimation. We also note that in a few cases (around 6 s in the lower subplot of Figure 5), the analytical model actually predicts a velocity in the wrong direction. We suspect this may be due to unmodeled non-linear stiffness properties of the robot because at this moment in time (6 s), the robot is near its upper joint limit of 1.5 radians in both θ and ϕ (see Figure 6).

Our primary observation from Figure 6 is the large steady-state offset caused by plastic deformation on the real hardware resulting in a non-zero equilibrium configuration in the joint angles θ and ϕ, but which is not present in the analytical model and the surrogate DNN. The error model is able to eliminate most of the offset and track the hardware data well. While there are some small transient dynamics in the hardware data that were not learned by the error DNN (e.g., the 4, 6, and 18 s marks for θ or 4, 6, and 16 s marks for ϕ in Figure 6), the error DNN prediction performance is clearly superior to that of either the analytical or surrogate DNN.

**Figure 7:**
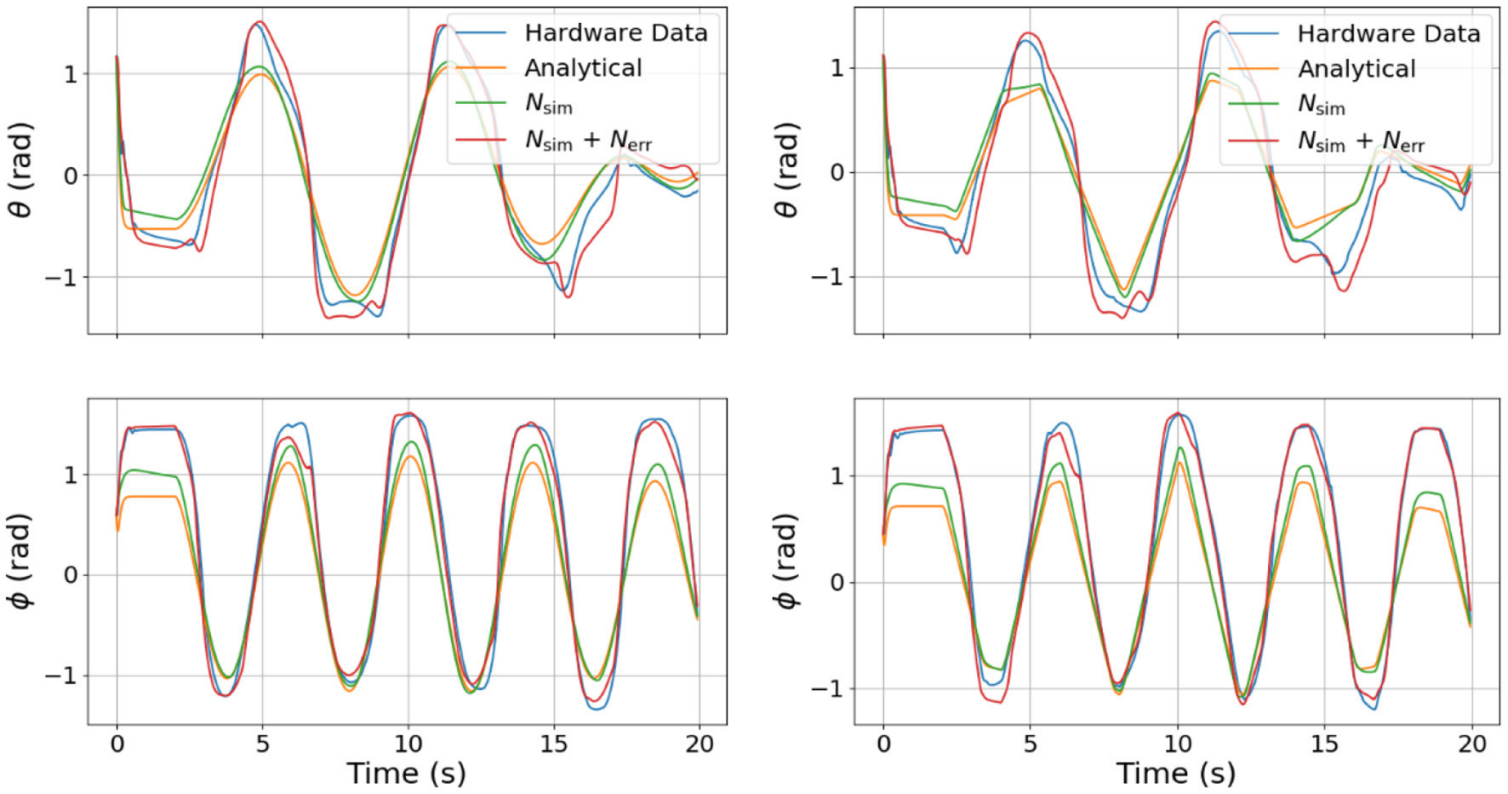
Comparison of joint angle dynamics between the four different models used on different test signals. The left column is in response to sine waves in pressure commands and the right column is in response the ramp inputs in pressure commands. Note that the combined DNN, although not trained on sines and ramps, still predicts unmodeled dynamics.

In an effort to explore the transferability of our model, we also tested our DNNs, sending pressure trajectories that were not used for training (e.g., sine waves and ramps). The results of this experiment are shown in Figure 7. The left column is the model prediction using sine wave pressure inputs and the right column is the model prediction using ramp pressure inputs. It is interesting to note that the error DNN (*N_err_*) learned to compensate for some coupling clearly visible in θ, as well as some offsets in both θ and ϕ that are not captured by the analytical model or the surrogate DNN (*N_sim_*) alone.

## 3. Control

In this section, we present our control algorithm and our findings based on several experiments in simulation and on hardware.

### 3.1. Non-linear Evolutionary Model Predictive Control

Non-linear evolutionary model predictive control (NEMPC) was developed as a real-time control algorithm for high degree of freedom (DoF) robot platforms. A variant of model predictive control (MPC), NEMPC utilizes an evolutionary algorithm to solve the MPC optimization. By using an evolutionary algorithm, it is able to approximate a global minimum (as opposed to an exact local minimum) because it explores more of the solution space than local optimization methods. Extensive implementation details can be found in papers by Hyatt and Killpack (2020) and Hyatt et al. (2020b).

The implementation of NEMPC in this work differs from the work in Hyatt and Killpack (2020) in that the algorithm no longer mutates every child generated during mating. With some probability *P_mutate_*, children are selected for mutation. Those children have each of their genes perturbed by a uniform distribution on the interval (−σ, σ). This allows the search to refine individual trajectories while still preserving others.

For this paper, we implement the typical quadratic cost function formulation used in other MPC schemes with one small modification that places a cost on the change in inputs (i.e., Δ*u*_*t*_ = *u*_*t*_−*u*_*t* − 1_) as opposed to *u*_*t*_ itself. This forces NEMPC to generate more conservative solutions which in turn, cause pressure to vary more smoothly over time. Note that the cost on change in inputs is a competing optimization objective with position tracking and requires some tuning of *Q* and *R* to achieve good tracking performance while also maintaining smooth input trajectories. The optimization is formulated as

(7) $$\begin{array}{l} minimize\;J=\sum_{t=0}^{T-1}[{\left(x_{t}-x_{goal}\right)}^{⊤}Q(x_{t}-x_{goal})\\ \;+\Delta u_{t}^{⊤}R\Delta u_{t}]\;+{\left(x_{T}-x_{goal}\right)}^{⊤}Q_{f}(x_{T}-x_{goal})\\ \;w.r.t.\;u_{t},\;\forall t\in 0,1,\ldots ,T\\ \;s.t.\;x_{min}\leq x_{t}\leq x_{max},\;\forall t\in 0,1,\ldots ,T\\ \;u_{min}\leq u_{t}\leq u_{max},\;\forall t\in 0,1,\ldots ,T\\ \;x_{t+1}=x_{t}+N,\;\forall t\in 0,1,\ldots ,T \end{array}$$

where

(8) $$\begin{array}{l} N=N_{sim}\left(x_{t},u_{t}\right) \end{array}$$

or

(9) $$\begin{array}{l} N=N_{sim}\left(x_{t},u_{t}\right)+N_{err}\left(x_{t},u_{t}\right). \end{array}$$

In Equation (7), *J* is a scalar representing the cost of a given input sequence, *T* is the simulation horizon over which that input series is applied, and *Q* ∈ ℝ^8×8^, $Q_{f}\in {\mathbb{R}}^{8\times 8}$ and *R* ∈ ℝ^4×4^ are diagonal weighting matrices penalizing error, error at the final time step of the horizon, and actuator effort, respectively. *x*_*t*_ represents the state vector and *u*_*t*_ is the input vector. *x_goal_* is the commanded robot state. *Q* and *Q*_*f*_ are weighted such that the only values of *x_goal_* that contribute to the cost *J* are the position and velocity states. The variable *N* is a placeholder for the DNN that NEMPC uses. For the case using the surrogate DNN defined in section 2.2, NEMPC enforces the constraint given in Equation (8). For the combined case defined in section 2.4, NEMPC uses the constraint given in Equation (9).

At each time step, the optimizer is allowed to take a single step toward the optimum (or one generation of the genetic algorithm). NEMPC then returns the input associated with the lowest cost member of the population for the current time step, which is applied to the hardware system. As soon as that command is sent, NEMPC takes another step toward the optimum, given new measurements of the robot's state. The fact that the previous time step's population is used to warm start the next optimization causes the algorithm to converge quickly.

As a practical note, the tuned weights in *Q* corresponding to the pressure states are 0 because we are not trying to follow a pressure trajectory or specify stiffness. This allows NEMPC to find any valid set of pressure states that will enable tracking of desired velocity and positions. Positions are weighted heavily and velocities relatively lightly.

The introduction of a DNN as NEMPC's internal model of the plant is a key component that enables NEMPC's execution at real-time speed and the evaluation of an entire population of solutions in batches. This allows a large graphics processing unit (GPU) to simultaneously evaluate all 1,500 potential input series at any given time step. In our work, we are able to control the eight state soft robot continuum joint at a rate of 100 Hz with a time horizon of 0.1 s.

### 3.2. Simulation Experiment

To validate the efficacy of the NEMPC controller, a simulated experiment is run using the analytical model of the soft robot continuum joint as the plant, and the surrogate DNN as the internal control model of the system. As in later hardware experiments, NEMPC is fed a reference trajectory in θ and ϕ, and calculates a set of reference pressures $u_{t}^{*}$ which are then applied to the dynamic system (simulated with the analytical model in this case). This experiment is not run in real time, due to the computational time required to numerically integrate the analytical model of the robot.

### 3.3. Simulation Results

**Figure 8:**
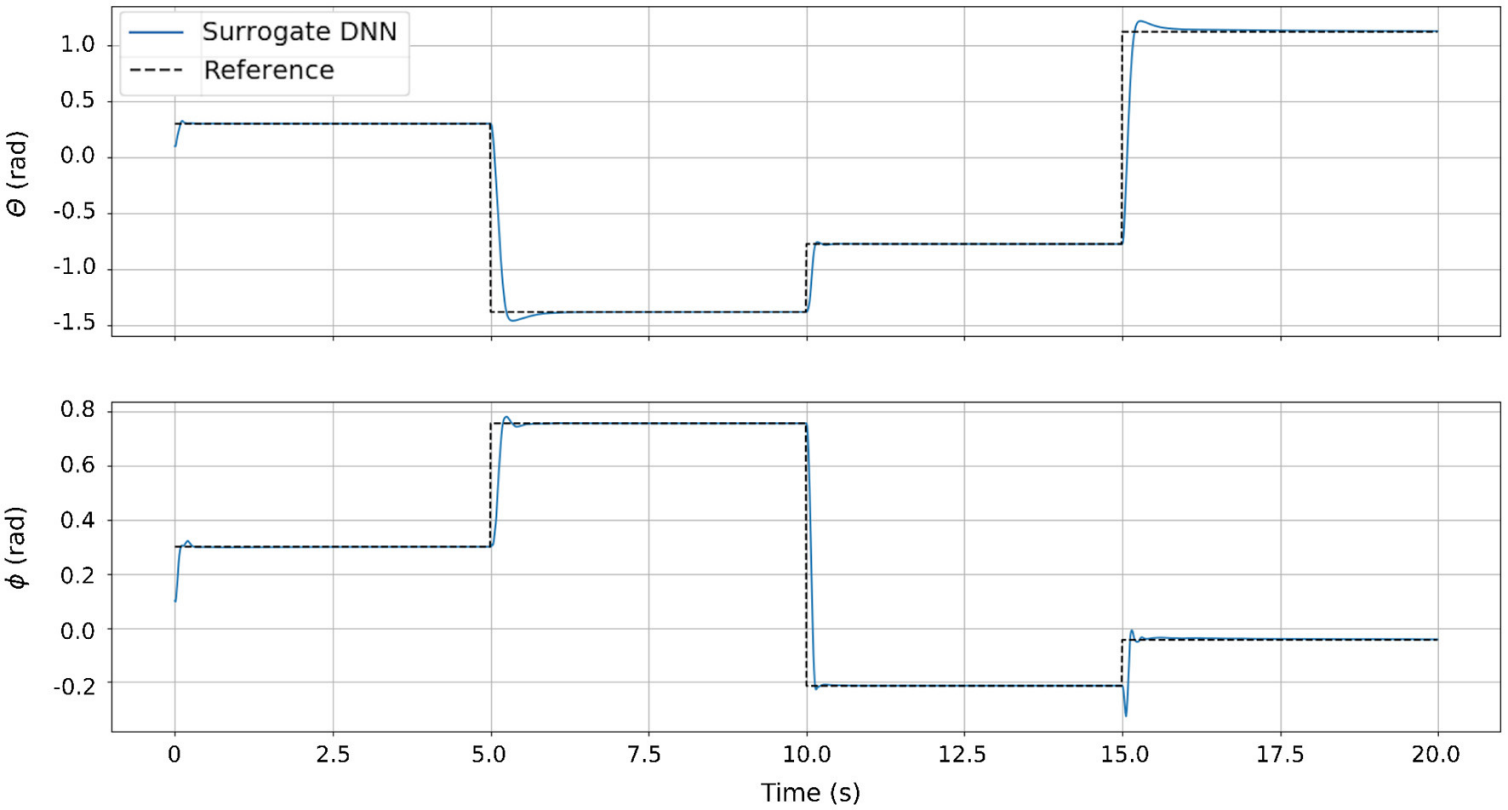
The control performance of NEMPC in simulation. For this trial, the analytical model of the soft robot continuum joint acts as the plant, while NEMPC uses the surrogate DNN as its internal model of the system. Since the surrogate DNN is a very good approximation of the analytical model of the robot, the controller has a near perfect model of the plant, resulting in excellent tracking performance.

The results of the simulation experiment can be seen in Figure 8. Since the surrogate DNN is a good approximation of the simulated robot, NEMPC is able to find near-optimal solutions with relative ease. From these results, we see that Non-linear Evolutionary Model Predictive Control is capable of generating excellent control inputs for a system that is well-approximated by a surrogate DNN. However, when NEMPC is used to control the hardware with a surrogate DNN, the results are much worse because the surrogate DNN is a poor approximation of the dynamics for the real hardware (see section 3.4).

### 3.4. Hardware Experiments

After validating NEMPC's performance in simulation, we evaluate the performance of NEMPC while controlling the soft robot continuum joint, following a reference trajectory in θ and ϕ. This experiment is run twice, once while NEMPC's internal model of the robot is represented by the surrogate DNN (*N_sim_*), and once while NEMPC's internal model is represented by the combined DNN (*N_sim_* + *N_err_*).

**Figure 9:**
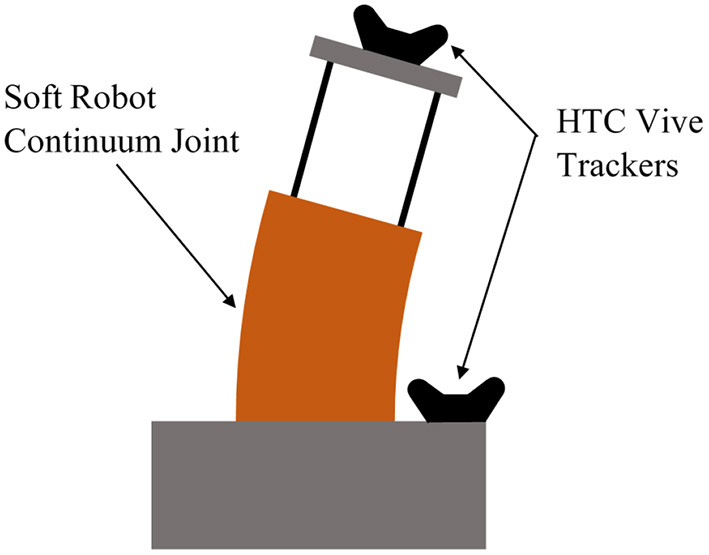
Diagram of the experimental setup for the hardware experiments. Also illustrated here is the inherent plasticity of the robot, resulting in a variable offset in θ and ϕ. Over time, the plastic in the pressure chambers deforms and causes the robot to have an equilibrium configuration that is not vertical.

We use two HTC Vive Trackers rigidly attached to the robot base and tip in order to measure joint angles (θ and ϕ) in real-time (see Figure 9), while the joint velocities ($\overset{∙}{\theta }$ and $\overset{∙}{\phi }$) are numerically differentiated from the angle measurements. The pressures in each of the robot's four chambers are measured by onboard sensors and controlled by an embedded high-frequency PID controller. All of this data is packaged and published via the Robot Operating System (ROS) at 400 Hz to a separate computer on the network with an 8 core Intel Xeon E5-1620 CPU and an NVIDIA GeForce GTX 1080 Ti GPU, which is dedicated to running the NEMPC algorithm. As shown by Thompson et al. (2020), the hardware requirements for major deep learning papers have increased quickly with time, so we believe that our single-GPU setup is relatively inexpensive and computationally cheap.

**Figure 10:**
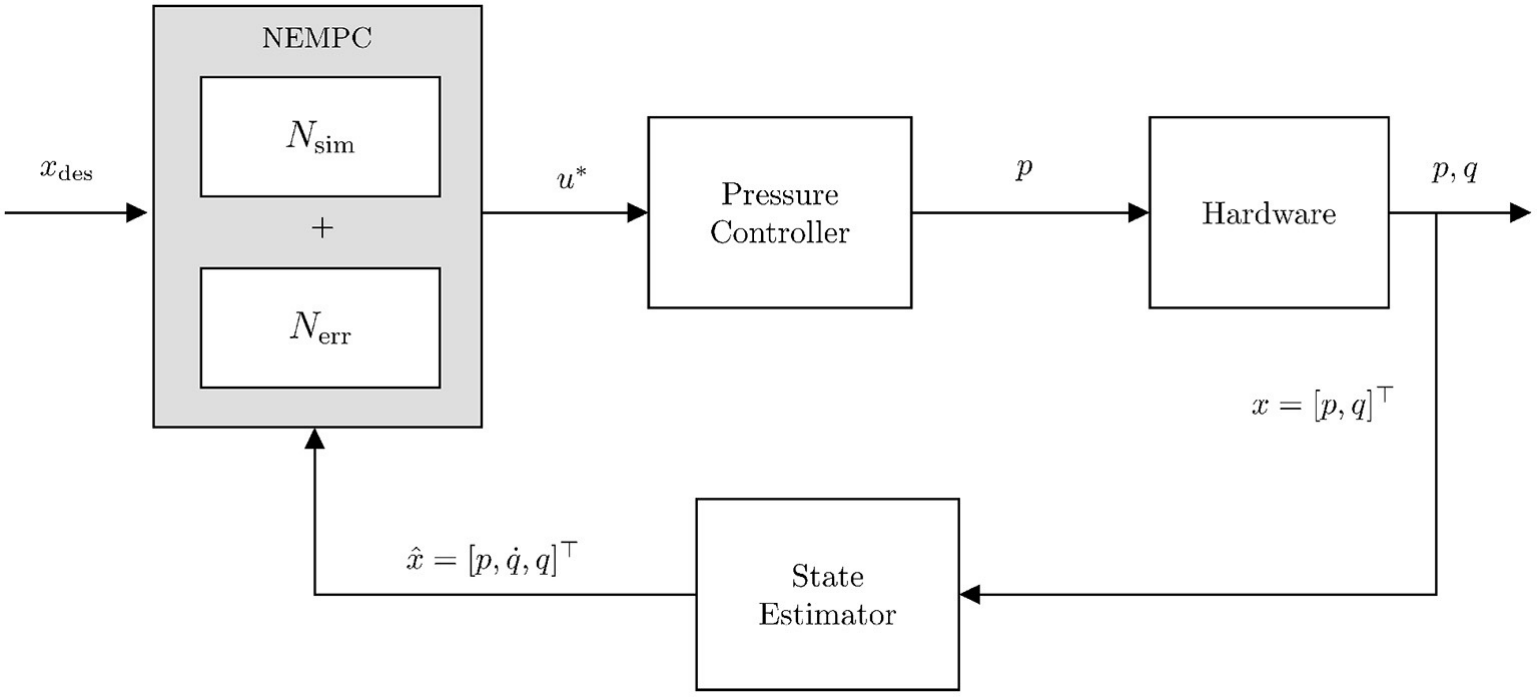
Control diagram for running NEMPC in conjunction with the learned error model. u^*^ indicates the optimal input chosen by the controller. This input is sent to the embedded pressure controller and we measure pressures *p* and positions *q* directly, while estimating $\overset{∙}{q}$.

Figure 10 illustrates the process as a control diagram. The controller is given an *x_des_*(*t*) which is used in conjunction with the current state estimate ${\hat{x}}_{t}$ to calculate an optimal pressure command u^*^. This command is sent to the embedded PID pressure controller and then pressures and joint angles are measured directly.

### 3.5. Hardware Results

**Figure 11:**
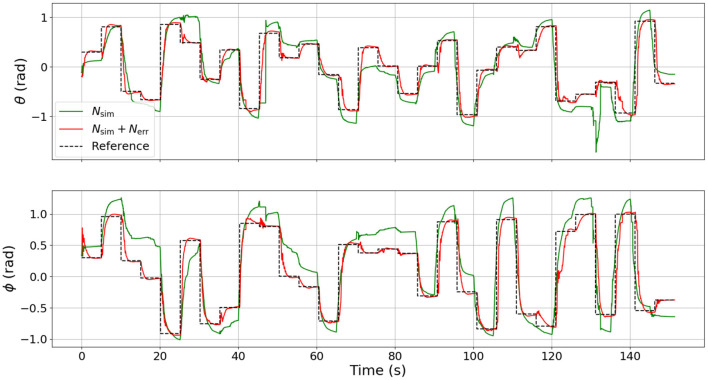
Comparison of tracking performance on the physical soft robot continuum joint while using the two categories of DNN model approximation. Note that the control performance of NEMPC while using the combined DNN contains much less steady-state tracking error than the control performance of NEMPC while using the surrogate DNN.

The results of the hardware experiments are presented in Figure 11. When the surrogate DNN is used as NEMPC's internal model to control the soft robot hardware, NEMPC struggles to follow the desired path for θ and ϕ. This behavior is likely due to the surrogate DNN's poor approximation of the hardware dynamics, as evaluated in section 2.5. Evidence of this is found in the performance of NEMPC while internally simulating with the combined DNN.

When NEMPC controls the hardware while using the combined DNN as its internal model, the reference tracking performance shown in Figure 11 improves significantly. With a more accurate internal model, NEMPC is able to generate solutions that better account for factors, such as the robot's plasticity (e.g., non-zero equilibrium configuration), hysteresis, and increased stiffness and damping near joint limits. This results in a much lower steady-state offset, and more rapid convergence in some cases.

Quantitatively, the reference tracking behavior of NEMPC can be measured through a statistical analysis of the tracking error for each experiment. A statistical comparison of NEMPC performance can be found in Table 2. The mean tracking error decreased from 0.378 to 0.182 rad, a 52% decrease. The median tracking error decreased by almost an order of magnitude. Of particular note is the difference in integral of the time-weighted absolute error (ITAE) for each trial. This measure penalizes errors that persist over time, and allows a controller to be slightly less aggressive, as long as it converges and stays close to its target. The ITAE is calculated for each step input individually, summed over the whole series of step inputs, then recorded. As seen in the table, NEMPC with the combined DNN greatly outperforms NEMPC with the surrogate DNN in regards to ITAE, in part due to its lack of significant steady-state error. The surrogate DNN could be helped by the addition of an integrator to the controller, as done in previous work with NEMPC by Hyatt and Killpack (2020).

**Table 2:** Comparison of control performance of NEMPC with error compensation vs. NEMPC without error compensation.

	**ITAE**	**Mean tracking error**	**Median tracking error**	**Execution time**
*N_sim_*	128.3496 rad^2^ s	0.37820 rad	0.31736 rad	0.0006 s (464x)
*N_sim_* + *N_err_*	21.5252 rad^2^ s	0.18180 rad	0.03676 rad	0.0009 s (287x)

*The integral of the time-weighted absolute error (ITAE) is a performance measure often used in tuning control algorithms, and is a measure of convergence for a given trajectory. The execution time for a single time step is listed in seconds as well as a multiplier of many times faster the DNN execution time was compared the analytical model implemented in C++*.

What is most impressive in this case is that by incorporating the combined DNN with NEMPC, we achieve very low steady-state error with no integral control at all. All of our prior work (and most of the soft robot control literature) has required some sort of integral or adaptive control to compensate for this steady-state error (see Hyatt et al., 2020a for an example of model-reference adaptive control (MRAC) which essentially exhibits integral action to achieve low steady-state error).

**Figure 12:**
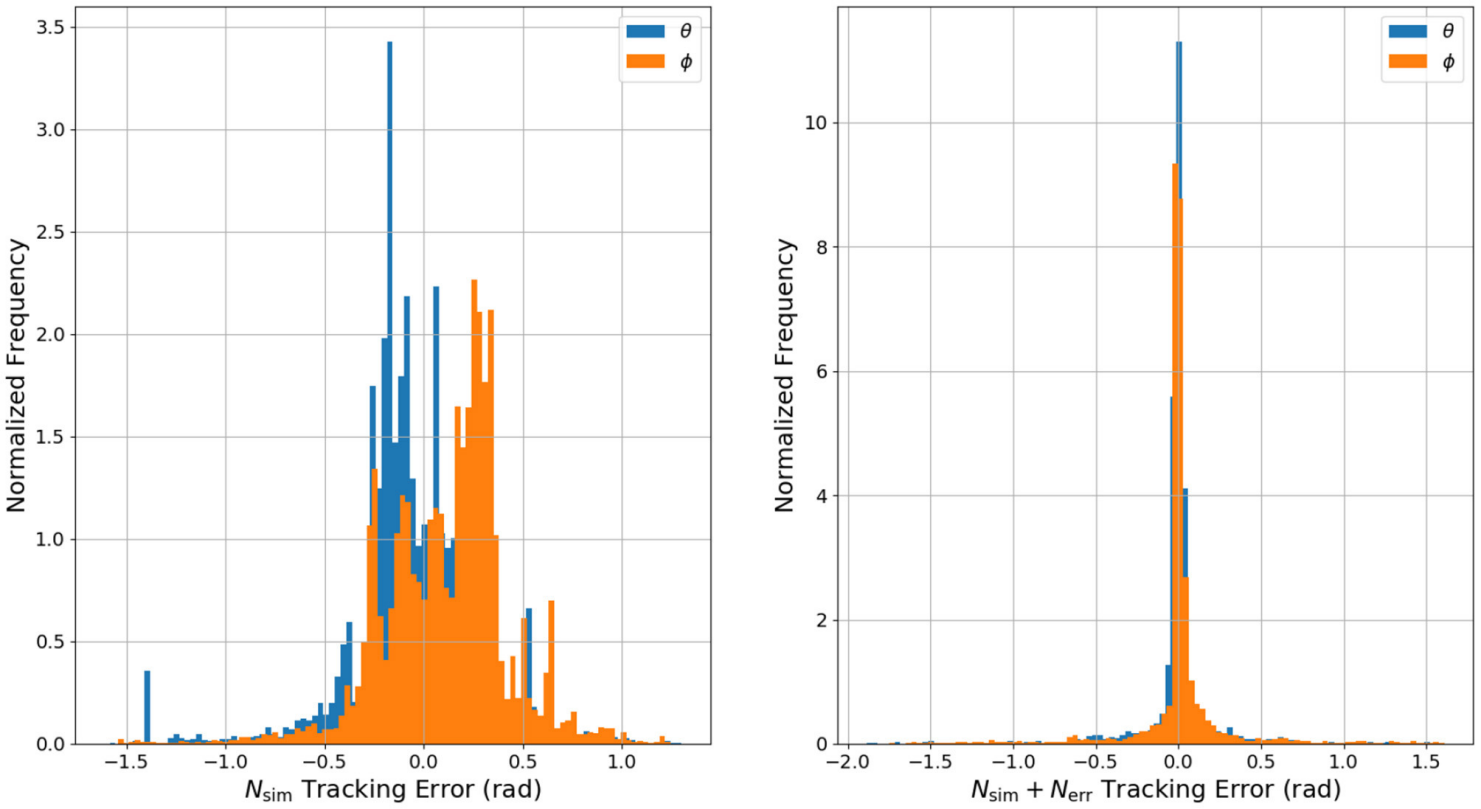
A histogram of the normalized frequency of θ and ϕ tracking error in the hardware experiments. Note that the data gathered while using the surrogate DNN for control has θ error and ϕ error that is biased in both directions away from zero. This is a result of the surrogate DNN's lack of information regarding the offsets in θ and ϕ at equilibrium. Also note the difference in y axis scaling for both histograms.

The implementation of an integrator could help reduce steady-state tracking error for the surrogate DNN controller, but the control would still suffer from overshoot and generally poor performance. The mean, median, and standard deviation of the tracking error would likely remain indicators of the surrogate DNN's relatively poor performance. To visualize the insights offered by the mean, median, and standard deviation of the tracking error, Figure 12 presents a histogram of the normalized frequency of error for each of the two experiments on hardware. Visible in the plot for the surrogate DNN is the angle offset due to the robot's non-zero equilibrium configuration. The surrogate DNN causes NEMPC to tend toward negative error in θ and positive error in ϕ. When the error model in the combined DNN is introduced, both θ and ϕ error are pulled toward zero, becoming uni-modal and more normally distributed. Overall, the combined DNN is a much better approximation of the robot's dynamics, allowing NEMPC to follow the given reference trajectory much more effectively, even with fast changes (step inputs) in the commanded changes for ϕ and θ.

**Figure 13:**
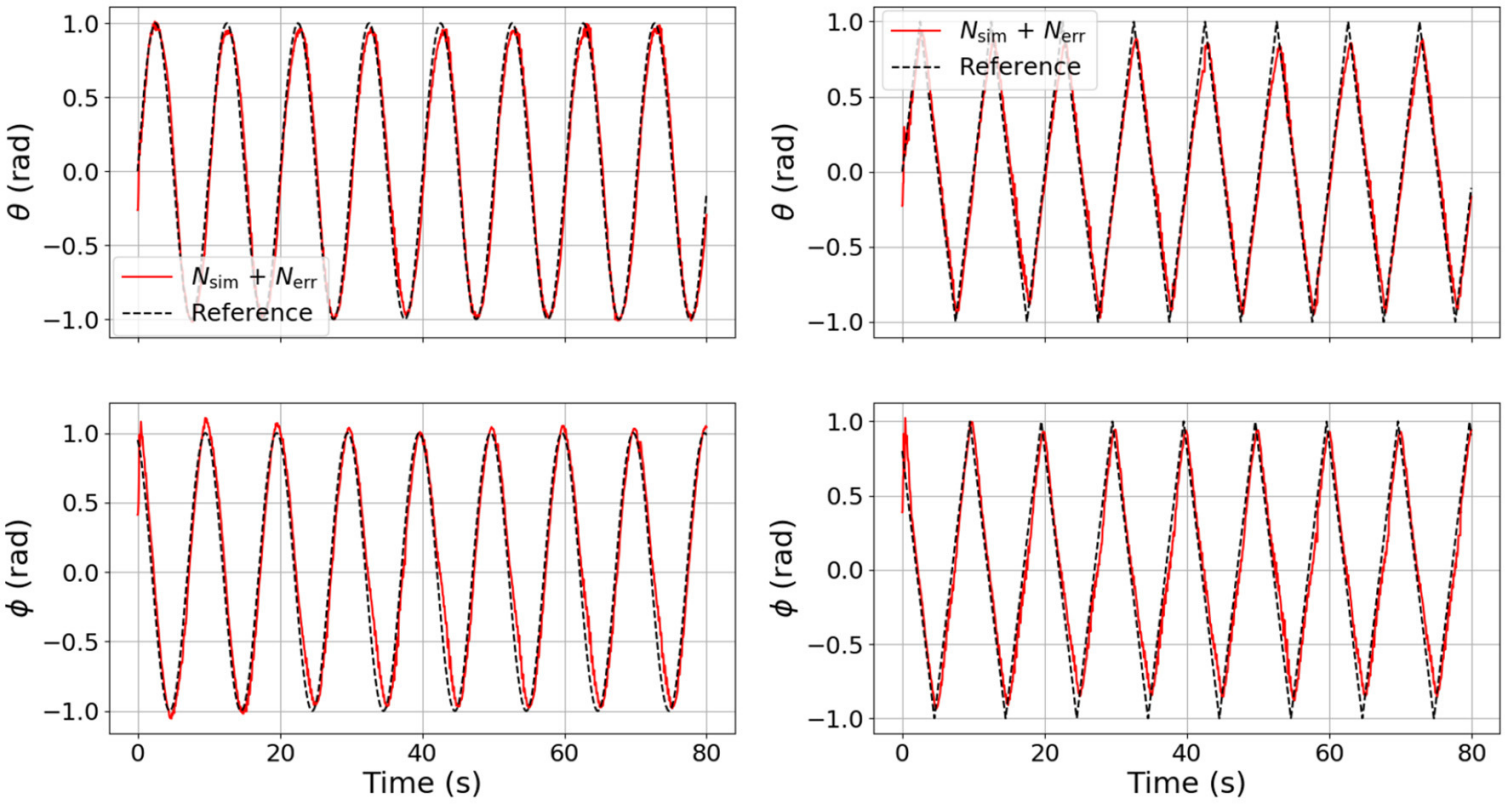
Comparison of tracking performance on sine (left column) and ramp (right column) test signals using the *N_sim_* + *N_err_* DNN configuration for control. Note that although both DNNs were trained solely on step inputs, the models are able to generalize well to other types of signals.

To validate that the combined DNN can be used for control trajectories other than step inputs, we conducted two more experiments: one for tracking sin waves in ϕ and θ and a second for tracking ramps in ϕ and θ. The results can be seen in Figure 13. From these figures, it is apparent that the training data consisting of only step inputs is enough for the DNN to accurately predict the performance of the robot while tracking other wave forms. There is a nominal amount of phase lag in both cases, but this is expected because, in our implementation of NEMPC, *x_goal_* for the entire prediction horizon remains constant while the waveform continuously changes. This could be overcome (without changing our formulation at all) by simply allowing NEMPC to use a continuous *x_goal_* trajectory instead of a single constant value which we used.

## 4. Conclusions and Future Work

In this work we demonstrate that significant model and control improvement is possible through a data-driven deep learning approach. Our approach does not require specialized expertise or any assumptions about the form of the model. As a result, this method is generally applicable to any model-based control problem where the plant dynamics are highly uncertain or only partially known.

Additionally, because our approach is rooted in a physics-based analytical model and our error DNN only needs to learn relatively small adjustments, the error DNN can be smaller, faster, and train with less data than would be required if we took a completely model-free learning approach. This is especially beneficial when gathering training data on hardware is dangerous or expensive, as is often the case in the field of robotics (albeit less so for many soft robots).

In future work we hope to improve DNN accuracy, including using a state buffer. Currently, the DNN state transition model can only see the current state and commanded pressures–in other words, we assume that the state transition model is a first-order Markov process. If hysteresis and other non-linear, state-dependent phenomenon are present, then performance may improve by including a buffer of the last *n* states. This time sequence data could be leveraged by a fully-connected network, or some kind of recurrent neural network (RNN). However, this approach may slow the evaluation of the network.

An important preliminary result, though not discussed in-depth in this paper, is that the model and controller were sensitive to the frequency content in the data used for training. The effects of this were significant, but are currently poorly understood. However, we have presented evidence that using square waves to explore and learn the state space is an efficient method because the trained models generalized relatively well to sine waves and ramps. We also note from our experiments that the inverse relationship is not true; models trained on sine waves and ramps generally did not perform well when tested on step inputs. We believe this is because square waves excite more dynamic modes than sine waves or ramp inputs in pressure. Further exploration into deep learning dynamics in a generalized fashion could be valuable as future work, especially in regards to specifically learning frequency content and modes of a dynamical system. This could produce even higher fidelity models. A downside to our approach is that if anything causes the plant dynamics to change, a small period of retraining would be required to maintain model fidelity. Future work could include a learning approach which allows the platform to continuously learn an error model online. Additionally, we recognize that exploring the state space randomly to gather training data is not always possible on some hardware platforms. Future work could include an exploration of how learning from a safe subspace of the state space can generalize to control over the entire reachable state space.

## Data Availability Statement

The raw data supporting the conclusions of this article will be made available by the authors, without undue reservation.

## Author Contributions

CJ contributed a general problem formulation, collected the training data, and ran the experiments. TQ contributed with NEMPC controller improvements and running experiments. TS contributed DNN structures and training methods. CJ, TS, and TQ contributed equally to writing the paper. DW and MK assisted in developing the methodology and in advisory roles. All authors contributed to the article and approved the submitted version.

## Conflict of Interest

The authors declare that the research was conducted in the absence of any commercial or financial relationships that could be construed as a potential conflict of interest.
